# Multi-class BCGA-ELM based classifier that identifies biomarkers associated with hallmarks of cancer

**DOI:** 10.1186/s12859-015-0565-5

**Published:** 2015-05-20

**Authors:** Vasily Sachnev, Saras Saraswathi, Rashid Niaz, Andrzej Kloczkowski, Sundaram Suresh

**Affiliations:** 1 Department of Information, Communication and Electronics Engineering, Catholic University of Korea, Bucheon, Republic of Korea; 2 Battelle Center for Mathematical Medicine at The Research Institute at Nationwide Children’s Hospital; currently at Sidra, Medical and Research Center, Doha, Qatar; 3 Department of Medical Informatics, Sidra Medical and Research Center, Doha, Qatar; 4 Battelle Center for Mathematical Medicine at The Research Institute at Nationwide Children’s Hospital; Department of Pediatrics, College of Medicine, The Ohio State University, Columbus, USA; 5 School of Computer Science, Nanyang Technological University, Nanyang, Singapore

**Keywords:** Global cancer map, Cancer biomarkers, Binary coded genetic algorithm, Extreme learning machine, Hallmarks of cancer

## Abstract

**Background:**

Traditional cancer treatments have centered on cytotoxic drugs and general purpose chemotherapy that may not be tailored to treat specific cancers. Identification of molecular markers that are related to different types of cancers might lead to discovery of drugs that are patient and disease specific. This study aims to use microarray gene expression cancer data to identify biomarkers that are indicative of different types of cancers. Our aim is to provide a multi-class cancer classifier that can simultaneously differentiate between cancers and identify type-specific biomarkers, through the application of the Binary Coded Genetic Algorithm (BCGA) and a neural network based Extreme Learning Machine (ELM) algorithm.

**Results:**

BCGA and ELM are combined and used to select a subset of genes that are present in the Global Cancer Mapping (GCM) data set. This set of candidate genes contains over 52 biomarkers that are related to multiple cancers, according to the literature. They include APOA1, VEGFC, YWHAZ, B2M, EIF2S1, CCR9 and many other genes that have been associated with the hallmarks of cancer. BCGA-ELM is tested on several cancer data sets and the results are compared to other classification methods. BCGA-ELM compares or exceeds other algorithms in terms of accuracy. We were also able to show that over 50% of genes selected by BCGA-ELM on GCM data are cancer related biomarkers.

**Conclusions:**

We were able to simultaneously differentiate between 14 different types of cancers, using only 92 genes, to achieve a multi-class classification accuracy of 95.4% which is between 21.6% and 38% higher than other results in the literature for multi-class cancer classification. Our findings suggest that computational algorithms such as BCGA-ELM can facilitate biomarker-driven integrated cancer research that can lead to a detailed understanding of the complexities of cancer.

**Electronic supplementary material:**

The online version of this article (doi:10.1186/s12859-015-0565-5) contains supplementary material, which is available to authorized users.

## Background

Somatic or genetic mutations in key regulatory genes may cause the molecular machinery to lose control over the regulation of cell proliferation, differentiation and death that can in turn lead to clonal proliferation, causing cancer. Identification of cancer through morphological features of tumor cells has serious limitations since similar histopathological appearances can imply various clinical and risk conditions. Recent studies in cancer genomics have created a body of knowledge that has facilitated better understanding of the complexities of cancer. Advances in molecular diagnostics have helped to make cancer classification that is more objective and precise. The complexity of cancer can be coded in terms of underlying principles that determine the transformation of normal cells to cancer cells [1,2].

Biomarkers are measured characteristics of biological conditions that can indicate favourable or adverse conditions present in cells. Advances in cancer research have revealed that mutational oncogenes and tumor suppressor genes are molecular markers characteristic of cancer. The application of computational methods to identify biomarkers that encode these cancer causing changes can provide clinicians with a valuable tool that could lead to advances in the understanding, treatment and prognosis for cancer.

Microarray data typically consists of thousands of gene features with only a few hundreds of samples. Computational biologists have applied Genome wide association studies using advanced statistical and bioinformatics techniques to better understand the etiology of cancer. Several studies in gene selection and classification methods have used the frequently used Global Cancer Mapping (GCM) [3] microarray gene expression and other cancer data sets [4-12]. Other improved and efficient methods include genetic algorithm for gene selection combined with SVM and fuzzy neural networks [13,14].

In our previous publication, an integer coded genetic algorithm and Extreme Learning Machine (ICGA-ELM) [15] multiclass approach was used. Other hybrid methods include particle swarm optimization (BPSO) and genetic algorithm (CGA) [16], an ensemble correlation-based algorithm with support vector machine [17] and the top scoring genes (TSG) algorithm [18] among many other studies.

The objective of this study is to select the best set of features (genes) that can *simultaneously* classify different types of cancers accurately and to help identify biomarkers. The Binary Coded Genetic Algorithm (BCGA) combined with the neural network based Extreme Learning Machine (ELM) is used to obtain high classification accuracy. BCGA-ELM is tested primarily on the GCM data set along with several other cancer data sets. These results are compared to popular classification methods using the Weka software [19]. BCGA-ELM compares or exceeds other algorithms (in literature) in terms of accuracy. Over 50% of the genes selected by BCGA-ELM are identified (through IPA analysis) as cancer-related biomarkers that are closely associated with the hallmarks of cancer [1,2].

## Methods

Several multi-class and binary class microarray data sets are used in this study. Global Cancer Map (GCM) is primarily used in this study to illustrate the capabilities of the BCGA-ELM algorithm in selecting cancer related biomarkers and in obtaining high classification accuracy. Other cancer data sets are included in this study to show the robustness and generalization capabilities of BCGA-ELM in selecting meaningful biomarkers that can achieve high accuracy, irrespective of the algorithms that are used for classification.

### Data

**Figure 1:**
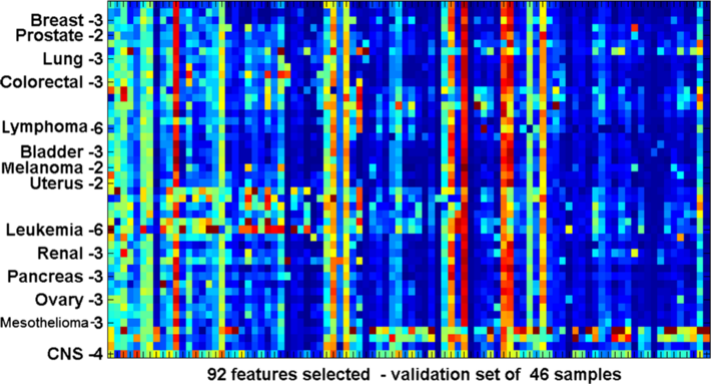
Gene expression for 92 features, selected by BCGA-ELM from GCM dataset (for one of the validation sets of 46 samples). The horizontal bars for each of the 14 different types of cancers show differentiated gene expression for different cancers, notably for Lymphoma, Leukemia and CNS, where broad horizontal bars that separate different types of cancers are seen distinctly. The x-axis represents the 92 genes while the y-axis represents the samples for each type of cancer (see Figure 1 and Additional file 1: Table S2 for gene names and descriptions).

GCM is an oligonucleotide microarray data obtained from solid tumors of epithelial origin [3]. GCM data is characterized by a large feature set with a small number of samples per class. 16063 features (genes) were extracted from 190 non-metastasized tumor samples spanning 14 different types (classes) of common cancers. 77 normal (control) samples were also included in this study for the binary classification of cancer vs. tumor. GCM data have a highly imbalanced data set, where sets of 144 randomly selected tumor samples that are used for training contain between 8 and 24 samples per class. The remaining 46 tumor samples that are used for testing contain between 2 and 6 samples each (Additional file 1: Table S1 and Figure 1). 20 cross-validated trials were conducted using randomized training and test sets, where similar sample distributions were maintained. From a total of 16063 genes, BCGA-ELM selects a small set of 92 genes that have the highest discriminatory power in classifying these cancers. BCGA-ELM was used for feature selection on other multi-class (Breast, Leukemia and Lymphoma [20] and binary class (CNS, Colon, DLBCL, GCM, Lung and Prostate [12]) cancer data sets. These data are also characterized by large feature sets with very few samples. The feature sets, number of samples and class information for these data sets are given in Table 1. Very small sets of features ranging between 11 and 73 genes are selected using BCGA-ELM, to classify these cancer sets with high accuracy.

**Table 1:** **Classification accuracy using four multi-class cancer data sets (GCM, Breast, Leukemia, Lymphoma) and six binary sets (CNS, colon, DLBCL, GCM, lung, prostate) show that performance of BCGA-ELM is superior and consistent over all these data sets. GCM multi-class has an accuracy of 95.4%, which is at least 21.6% higher than other methods given in the literature (although some of them use very small sets of genes)**

**Multi – class**					**Binary-class**					
**Data** **[** 3 **,** 12 **,** 20 **]**	**GCM**	**Breast**	**Leukemia**	**Lymphoma**	**CNS**	**Colon**	**DLBCL**	**GCM2**	**Lung**	**Prostate**
#Genes-initial set	16063	1213	999	4026	7129	2000	7129	16063	12533	12600
#Genes BCGA-ELM	92	30	11	27	17	27	18	73	11	72
# Samples	198	49	38	96	34	62	77	280	181	102
# Classes	14	4	3	5	2	2	2	2	2	2
Multi-class, Accuracy (%)					Binary-class, Accuracy (%)					
**BCGA-ELM**	**95.4**	**100**	**100**	**100**	**100**	**100**	**100**	**100**	**100**	**100**
	(^*^σ^2^ = 0.00083)									
**Weka packages** **[** 19 **]**										
LibSVM-linear	78.9	100	100	91.9	100	91.9	100	99.1	95.6	97.1
RBF Network	69.8	100	100	82.3	98.7	79.1	96.2	85.4	96.7	93.6
SMO	83.3	100	100	93.6	98.7	89.7	98.7	98.7	95.0	97.1
Naïve Bayes	78.6	100	97.1	72.6	93.5	60.0	81.9	73.1	97.8	92.7
Multiclass Classifier	85.3	100	100	93.6	97.4	93.5	99.8	99.7	94.5	98.8
Method	#Genes									
**ICGA-PSO-ELM [** 15 **]**	**42**	**88.3**	**91.2**	**100**	**97.0**	**100**	**-**	**-**	**-**	**-**
HC-k-TSP [8]	5 to 27	67.4	66.7	97.1	-	97.1	90.3	97.4	85.4	97.0
mul-PAM [9]	5 to 27	56.5	93.3	97.1	-	85.3	90.3	92.2	82.9	93.9
BMSF(highest) [10]	5 to 27	-	-	-	-	97.1	95.2	97.4	98.6	100
I-RELIEF(highest)[11]	5 to 27	-	-	-	-	88.4	82.3	95.1	96.1	91.2
LHR(highest) [12]	5 to 27	-	-	-	-	100	91.2	97.4	100	100

Current results show 4.2% improvement over our previous method using ICGA-ELM. All other multi-class and binary data sets are classified with 100% accuracy (shown in bold). Genes selected by BCGA-ELM (for all data sets) are classified using WEKA [19] machine learning package. These results are much lower for GCM multi-class data but are fairly consistent for other data sets compared to BCGA-ELM and other results in literature. (^*^σ is the variance).

Ingenuity Pathway Analysis (IPA®) is used to identify biomarkers among the selected candidate genes for four data sets (two each for multi-class and binary, as shown in Table 2). Ingenuity iReport® is used on 190 tumor samples and 77 normal samples, to compare aggregated tumor-normal gene expression signatures for each of the 92 genes. Ingenuity iReport® and IPA® use Ingenuity Knowledge Base® that has uniquely structured information related to cancer processes that are experimentally determined to be activated in cancer cells.

**Table 2:** **Selected Genes, biomarkers and activities related to hallmarks of cancer, as identified by IPA®, for four of the eight data sets are given here**

**Data**	**Names of selected genes**	**Biomarkers**	**Hallmarks of Cancer**
Breast - multiclass	CYC1, CYP2A6, DNASE1L3, EEF1D, EVI2A, GPM6B, HAS1, ICAM3, LAD1, LASP1, LEP, LMO4, LOC54157, LTBR, NAT1, PFKFB4, POU2F2, PPP1R1A, RBP1, TCEAL1, TDRD9, TIMP4	APOE, APOH, BMP7, CALB2, CLU, COL4A3, EGF, IL4, IL13, ITGAV, LEP, LGALS3BP, LTC4S, MAPK1, MED21, MTOR, PPARG, PTK2, RBP1, SLC29A1, SMAD4, STAT5B, TGFB1, THY1, TIMP4, TLR4, TNF, TREM1	Cell morphology, hematological system development and function ,cell-to-cell signalling and interaction, cell death and survival, cell-mediated immune response, cellular movement, cellular compromise, DNA replication, recombination, and repair, cell-mediated immune response.
Leukemia - multiclass	PHF15, SPTAN1, FOXI1, MPO, APOC1, CD33, PTX3, LSS, ZYX, ATBF1, WIT1	APOC1, CEBPA, JUNB, MPO, NOTCH3, PROC, ZYX	Hematological system development and function, immune cell trafficking, inflammatory response, tissue development, cellular function and maintenance, cell death and survival, cell morphology, tissue morphology, cell-to-cell signalling and interaction, cell-to-cell signalling and interaction, cellular function and maintenance, inflammatory response.
CNS - binary	RPS23,TAGLN2, MORC3, BNC1, CSF2, MCFD2, GTF2B, CORO2A, IGF2BP3, UCHL1, EEF1B2, CNR2, CSN1S1, ITIH3, (3 unknowns )	CCL2, CCL3L3, CD28, CD44, CDKN1A, CSF2, ETS1, FASLG, HTT, IGF2BP3, IL2, IL6, IL15, IL18,STAT3, TLR2, TNF, TREM1, UCHL1	cell cycle arrest, cell death and survival, cellular compromise, cell-mediated immune response, cell-to-cell signalling and interaction, cellular development, cellular growth and proliferation, angiogenesis, cellular movement.
DLBCL - binary	CIRBP, NID2, TRIB2, RPA2, TALDO1, CD28, ECH1, IQGAP2, CD37, CRYAA, ZFP36L1, PON1, CCR1, YWHAH, HLA-A (3 unknowns)	B2M, CALR, CCL5, CD28, CSF2, CCR1, CD28, CD37, CSF2, FLNA, GATA3, HLA-A, IFNG, IL2, IL5, IL2RG, OPRD1, PDCD1, PPARD, PTGER4, SLC7A5, TRAF2, YWHAH	cell-to-cell signalling and interaction, hematological system development and function, immune cell trafficking, inflammatory response, cell death and survival, cellular assembly and organization, cell cycle arrest, cell death and survival, DNA replication, recombination, and repair, cell death and survival, cellular assembly and organization, cell cycle arrest, cell death and survival, DNA replication, recombination, and repair, cell death and survival, cell morphology survival, cell morphology.

### Selection of candidate genes using BCGA-ELM

BCGA-ELM consists of the Binary Coded Genetic Algorithm (BCGA) and the fast learning Extreme Learning Machine (ELM) [21,22]. The genetic algorithm has the potential to search for the best solution and ELM is capable of accurately classifying sparse data [22].

Genetic algorithm (GA) was developed [23] to design and build artificial systems that mimic natural systems. GA that implements the wrapper method, [24,22], are widely used to solve complex feature selection problems. In a wrapper method, a machine learning algorithm (such as ELM) continually evaluates different sets of genes selected by the GA. This hybrid genetic algorithm implements different types of genetic operators, at different stages of the evolution process, to execute an effective search and provide the best solution. A complete survey of genetic algorithms for various complex optimization problems can be found in [25]. We give a brief description here.

**Figure 2:**
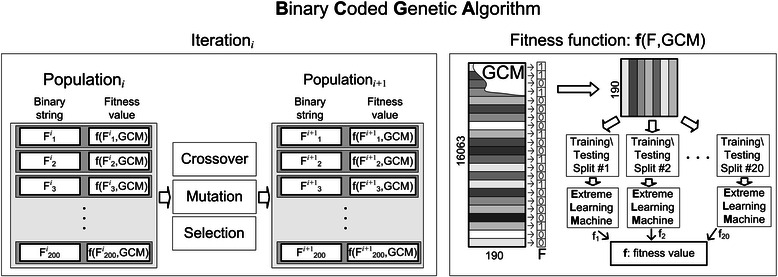
Framework of the proposed Binary Coded Genetic Algorithm, which is initialized with a randomly selected set of 200 solutions. These sets of genes undergo genetic operations such as crossover, mutation and selection, and are continually evaluated by ELM, until the termination criteria is met (maximum number of iterations or maximum classification accuracy). Computing fitness value *f*(F,GCM), where F is a binary string, GCM is a Global Cancer Map data base, *f* is fitness value computed using Equation 1.

The solution for our gene selection problem is coded as a binary string of length 16063, representing the total number of genes. A '0' in the string indicates exclusion of the gene in that position and a '1' represents inclusion of the gene (see Figure 2). In the initialization step, we generate 200 random binary strings (limited by our computational and time constraints) resulting in the first population of the 200 solutions. We have used normalized geometric ranking method given in [25,26] for the selection process. The number of chosen genes are randomly determined (between 20 to 200 genes) in each solution set. Each subset of features is used to compute a fitness value (see Figure 2) in each of these 200 solutions. A survival of fittest strategy is adopted where every string is evaluated during each iteration and the genes that represent the best fit (highest accuracy so far) are retained. Subsequently, probabilistic genetic operators (crossover or mutation) are used to create new solutions for the next generation, as shown in Figure 2. The hybrid crossover operator presented in this study generates four offspring for each pair of parents by uniform crossover and two point crossover operators. The most promising offspring of the four, substitute their parents in the population. We use the random mutation operator to ensure diversity in the population, in order to overcome the premature convergence and local minima problems. The fitness of the solution is determined by a higher mean testing accuracy obtained by the ELM, as given in equation 1.

1 $$ f=\left\{\begin{array}{c}\hfill {\overline{\eta}}_a + \frac{\omega_f}{{\displaystyle {\sum}_{i=1}^{16063}}{F}_i},\kern1.25em  if\ {\overline{\eta}}_a>d\ \hfill \\ {}\hfill {\overline{\eta}}_a\kern5.5em  otherwise,\hfill \end{array}\kern2.25em \right. $$

where, ${\overline{\eta}}_a$ is the mean validation accuracy from 20 random splits, ω_*f*_ is the cost of feature selection and *d* is expected accuracy. The sum in the denominator counts the number of 1’s in the string.

The data are divided into training (75%) and testing (25%) sets randomly. ELM classifier is used to compute training and testing accuracies. Random splitting and classification is processed 20 times on each of the 200 binary strings. Fitness value *f* (the mean of 20 testing accuracies) is computed using Equation 1. The final number of genes selected (92) is determined by the number of genes present in the solution set with the highest accuracy. A 20-fold cross-validation of the chosen gene set (represented by binary string) may guarantee a stable and robust solution for gene selection.

In our experiments we use ω_*f*_ = 1 and *d = 0.98* in Equation 1. The process of selecting the best genes continues during successive generations until the termination (convergence) criterion (maximum number of generations or maximum accuracy) is satisfied. In our experiments we use the following settings for GA: crossover probability 80%, mutation probability 20%, selection probability for normalized geometric ranking method is *q* = 1% over 50 generations. Through many iterations and evaluations, we arrive at a smaller set of 92 genes that satisfies our objective to obtain high accuracy.

The core of the feature selection approach is the ELM classifier, a fast learning algorithm, which is a single hidden layer feed forward neural [21]. In the ELM algorithm, the input weights connecting the input layer and hidden layer are chosen randomly and output weights are calculated analytically. ELM evaluates the genes selected by BCGA, in every iteration. The objective of the ELM classifier is to approximate the decision function f_*c*_ : *x* ^*t*^ → *y* ^*t*^ as accurately as possible. A comprehensive description of the ELM algorithm is given in [21]. The simple steps involved in the ELM algorithm can be summarized as follows:

- Given training samples and class labels (X*i*,*Yi*), select the appropriate activation function and number of hidden neurons.
- Randomly select the input weights *V*, bias *b* and calculate the output weights *W* analytically, where $W=Y{Y}_h^{\dagger }$ and ${Y}_h^{\dagger }$ is the Moore-Penrose pseudoinverse of matrix Y_h_.
- Use the calculated weights (*W*, *μ*, σ) for estimating the class label in the test set.
- The class label is estimated as the maximum value of *k* outputs ${\mathrm{y}}_i^k$.

2 $$ {\widehat{c}}_i=\begin{array}{c}\hfill \arg \kern0.5em  \max {y}_i^k\hfill \\ {}\hfill k=1,\ 2,\dots, C\hfill \end{array} $$

where *arg* function returns the class value with the maximum output.

ELM can be further improved through proper selection of ELM parameters (input weights, bias values, and hidden neurons). This is shown to influence the generalization performance [22,15] of the ELM multiclass classifier favourably by minimizing the error defined as:

3 $$ \left\{{H}^{*},{V}^{*},{b}^{*}\right\}=\begin{array}{c}\hfill \arg \kern0.5em  \min \left\{Y-T\right\}\hfill \\ {}\hfill H,V,b\hfill \end{array} $$

where *Y* is the observed class value and *T* is the calculated output value of the class, for a given set of hidden neurons *H* and input parameters *V* and *b*. The best weights and bias values for the ELM can be found using search techniques and optimization methods that are not very computationally intensive. These parameters are stored and used later on to determine the class of new samples.

In this paper we display an overall accuracy as a general measure of method performance. Overall accuracy is a ratio of number of correctly classified samples to total number of available samples.

## Results

### Discovery of biomarkers by BCGA-ELM

The BCGA-ELM algorithm selects the minimum set of 92 candidate features (from GCM data) that have the best discriminatory power to differentiate between 14 types of cancers, with 95.4% accuracy (where accuracy is the proportion of true results, both true positives and true negatives, among the total number of cases examined). Figure 1, illustrates the differential expression of these 92 genes for different types of cancers, for a set of 46 test samples. BCGA-ELM selects smaller sets of features, ranging between 11 and 73 genes, from 8 other cancer data sets which help to classify these cancers with high accuracy (see Table 1). These data sets with reduced features, give good results when tested using Weka [19] packages (using default parameters) illustrating the robustness and generalization capabilities of BCGA-ELM.

**Figure 3:**
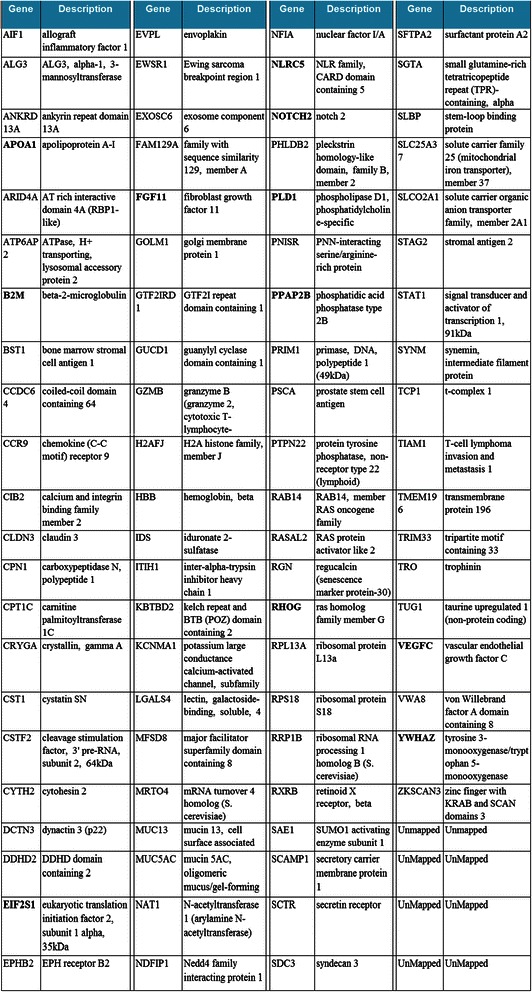
Gene names and description for 92 genes selected by BCGA-ELM. Some of the important genes implicated in signalling and metabolic pathways as determined by IPA® and iReport® analysis are in bold letters.

**Figure 4:**
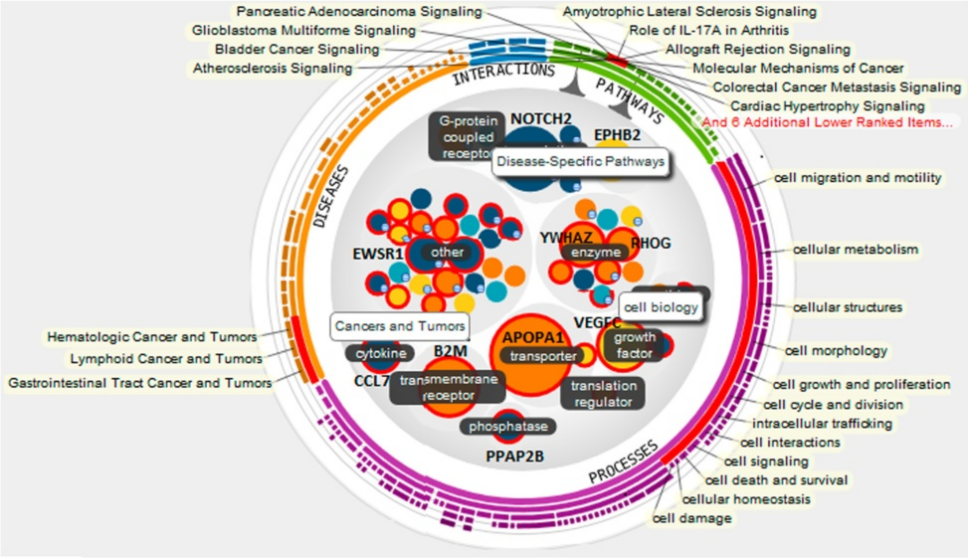
The genes that are involved in various cellular activities as indicated by iReport® analysis of the cancer vs. normal data analysis of 92 candidate genes (selected by BCGA-ELM) are displayed inside a wheel here. This figure was consolidated from several figures (given separately in the supplement) in order to show all cell activities in the same figure. Genes that are involved in cellular activities such as signalling, metabolism, growth, apoptosis, survival and proliferation, disease specific interaction and signalling pathways are listed here. This wheel displays the most important 52 candidate genes, where different colours and size of genes indicate various properties. The blue and green colours on the outside of the big circle represent interactions and pathways. The purple markings are for different processes and the orange outer circles are for different diseases. Genes are grouped under three major circles for diseases, interaction pathways and processes as indicated by light grey background circles. The size of the genes indicates the number of diseases/molecular functions/processes they are associated with. Gene circles are coloured according to their expression levels, which range between −3.304 to 1.637, where blue is for lower values and orange for higher values. The small blue circles on the south-east corner of the circular gene symbols indicate that these genes have isoforms. There are 103 pathways involving 20 differentially expressed genes (DEGs), 341 processes involving 40 DEGs and 79 diseases involving 29 DEGs. Some of these are illustrated here and fully listed in Additional file 1: Tables ~ S5 and S6. Genes related to particular types of cancers that are highlighted on the left of the figure are circled in red. APOPA1, NOTCH2, B2M and VEGFC seem to play major roles in these cancers. Genes responsible for cell death and survival are also given here.

An in-depth, insilico analysis of this data using IPA® and iReport® show some interesting results. This analysis indicates that over 52 of the 92 genes are determined to be significantly differentially expressed genes (DEGs). Figure 3 and Additional file 1: Table S2 give the full list of 92 genes with their gene names, description, fold-change, cell location, type of molecule and biomarker properties. Additional file 1: Table S3 lists the 52 differentially expressed genes. Top results based on ‘keyword search for cancer types’ show many of the pathways and diseases associated with the genes selected by BCGA-ELM (Additional file 1: Table S4). These genes are involved in 25 pathways, 66 biological processes, 29 diseases and 3 interactions (see Figure 4 and Additional file 1: Tables S5 - S6). Additional file 1: Table S7 shows the top 25 signalling and metabolic pathways in normal vs. cancer for the selected candidate genes. Additional file 1: Figure S3 shows the important genes involved in a network in breast cancer, overlaid with biomarkers, while Additional file 1: Table S8 shows the top molecules (biomarkers) implicated in Leukemia (as an example) as discovered by BCGA-ELM. IPA studies on the genes selected from the other eight multi-class and binary data sets yield several biomarkers for each data set. Table 2 lists the candidate genes, biomarkers and functions related to hallmarks of cancer for four of these sets.

## Discussion

### Performance Comparison of BCGA-ELM Classifier with Existing Methods

Table 1 gives the comparative analysis of results obtained using the BCGA-ELM approach for GCM and eight other data sets, We compare our results by running the same data under the Weka packages [19] and with other methods reported in the literature (a representative set). Most of the studies in literature are based on binary or quasi-binary (One Against All) classifications, while our method employs *simultaneous multi-class classification* of the data and gives high classification accuracy. The minimum number of genes required by each method to achieve maximum generalization performance is also given.

From Table 1, we can see that the proposed BCGA-ELM selects a minimum 92 genes (GCM) with a testing accuracy of 95.4%, which is 4.2% higher than our previous results. Our results show an increase of 21.6% over the original Ramaswamy et al. paper [3] for a smaller set of 92 genes, while other studies with small number of genes have accuracy that are less by 28 to 38% when compared to our results. The Weka [19] packages give accuracy that are lesser by 10 to 25.6% (for GCM) when compared to BCGA-ELM.

The accuracy for multiclass data sets Breast and Leukemia, with 30 and 11 features respectively, are 100% for BCGA-ELM and for the Weka algorithms (with a single exception for Leukemia which is 97.1% under Naive Bayes). The results are lower by 33.3% and 6.7% for HC-k-TSP and mul-PAM respectively (between 5 and 27 features) for Breast cancer while they are lesser by 2.9% for Leukemia. For Lymphoma (using 27 features), BCGA-ELM achieves 100% while the Weka packages yield between 72.6% and 93.64%. The lowest results are for Naive Bayes, which seems to be the general pattern for all data sets. We have given comparative results for other methods in the literature only when they are clearly stated as multi-class computations.

For the six binary data sets (CNS, Colon, DLBCL, GCM, Lung and Prostate) BCGA-ELM achieves 100% classification accuracy for all these sets, with reduced features ranging between 11 and 92 genes (see Table 1). The Weka results range on an average between 82.8% and 97.7% for these six data sets where the lowest result is 60% and the highest is 100% with an overall average of 93.1%. These results show the robustness and good generalization performance for the genes selected by BCGA-ELM. The results in the literature for these six binary data sets range on an average between 90% and 97.1%, where the lowest result is 82.3% and the highest is 100% with an overall average of 94.3% (except for GCM and prostate data sets, we have used a comparable number of genes in our study). Overall, BCGA-ELM exceeds all other classification algorithms in literature and in Weka, for all four multi-class and all six binary data sets that are used in this study, thus illustrating the superior capabilities of BCGA-ELM.

Although other studies in the literature given in Table 1 achieve similar or comparable accuracies, rarely do those studies follow up with the biological analysis of the selected genes that relate them directly to cancer. A comprehensive list of gene analysis relating selected genes to cancer pathogenesis is not seen in most of these studies. In Ramaswamy et al. [3], very few genes (4 out of 98) are identified as previously known biomarkers. In addition, they identify some signalling pathway targets that are statistically significant to certain types of cancers. In our previous work [15], we found a larger representation of genes that encode secreted proteins in our candidate sets, but no biomarkers were identified. The emphasis of this study is to illustrate that our algorithm is superior to other methods not only with respect to accuracy but is also capable of selecting features (genes) that are closely and directly related to hallmarks of cancer.

In addition to achieving high accuracy, this study highlights several biological properties and cancer specific biomarkers that relate 52 out of 92 of the GCM genes (more than 50%) to hallmarks of cancer (HC). To our knowledge, we have not seen such a large selection of biomarkers present in the candidate set of genes selected from the GCM dataset features (using computational methods). The remaining 40 genes, other than the 52 biomarkers that were identified by IPA® and iReport®, may be investigated further to determine if they are related closely to the pathogenesis of cancer. Similarly Table 2 also lists many of the biomarkers and functions for the genes selected by BCGA-ELM, from four of the other eight multi-class and binary data sets. These results show that BCGA-ELM is capable of selecting features that are highly involved in activities related to the hallmarks of cancer [1,2].

### Hallmarks of cancer related to the genes discovered by BCGA-ELM

Clinical and histopathological data are generally used to establish the diagnosis and treatment of cancer patients. Under difficult or advanced disease conditions, these data are not sufficient to make clear diagnosis or propose treatments. According to Hanahan and Weinberg [1,2], there are six underlying factors that are responsible for a cell being transformed from a normal state to a neoplastic cell, after which the cell ceases to be under the control of normal body processes. During this multi-step conversion process, the cancerous cell acquires several biological capabilities that constitute the hallmarks of cancer (HC).

Ingenuity Pathway Analysis® (IPA) and iReport® have identified 52 differentially expressed genes (DEGs), out of the 92 genes selected by BCGA-ELM, as known biomarkers that are closely related to the six hallmarks of cancer. This type of information can be used for the diagnosis and treatment of cancer. The expression changes were interpreted in the context of pathways, biological processes, disease phenotypes and molecular interactions. These hallmarks include cell processes such as proliferative signalling (HC1), developing resistance to cell death (HC2), immortalizing cells through replication (HC3), promoting growth of new blood vessels (vasculogenesis) to sustain tumors (HC4), invading healthier tissues (HC5) and promoting spread of cancer to other parts of the body (HC6). These activities include self-sufficiency in growth signal, insensitivity to anti-growth signals, tissue invasion and metastasis, limitless replicative potential, sustained angiogenesis and evading apoptosis.

**Figure 5:**
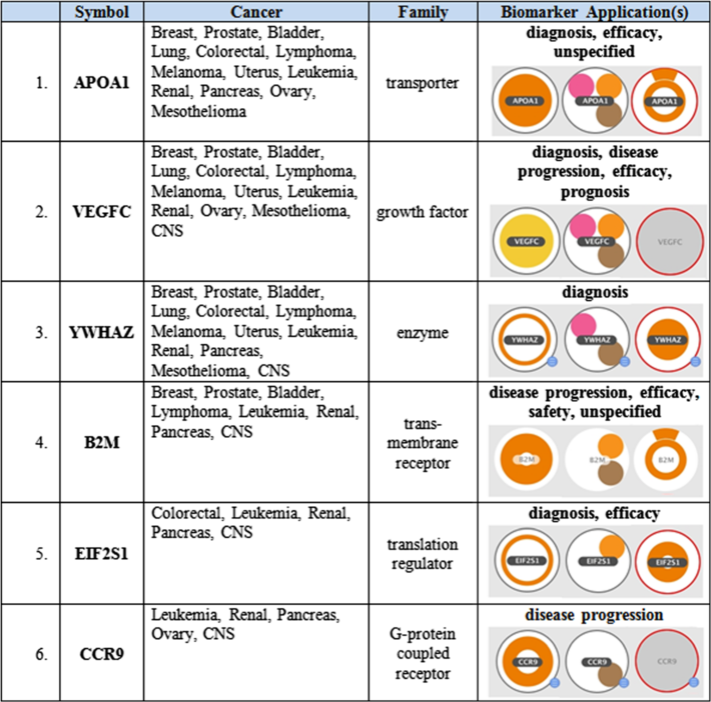
The top six of twelve biomarkers are listed in this table, with their family classification, such as transporters, growth factors, enzymes or regulators. Each biomarker is related to multiple cancers, with the top three biomarkers are related to almost all but one of the 14 cancers. The degree of filling of the circles denotes the number of processes in which the gene is involved in. The genes represented as filled circles, in the last column, under biomarker applications indicate the processes or disease related evidence, such as diagnosis, efficacy, disease progression, prognosis and safety. It can be seen that one biomarker can be active in multiple cancer classes, with APOA1 involved with all 13 cancers, except CNS. Similarly VEGFC is related to all but pancreatic cancer, while YWHAZ is related to all but ovarian cancer. These biomarkers are useful for diagnosis or determining the efficacy of drugs, while some of them are unspecified. Other colour coding in this figure are similar to those described in Figure 4.

Figure 4 shows genes that are involved in activities such as cellular metabolism, growth, death, survival and proliferation, among others. Additional file 1: Figure S1 shows genes that are responsible for cell death and survival. Figure 5 shows the top six of twelve biomarkers that were recognized by Ingenuity® IPA. The molecular family and the biomarker application for each gene is given. The biomarkers belong to several biological categories such as transporters, growth factors, enzymes, trans-membrane and G-protein coupled receptors and translation regulators. These biomarkers are used for several medical applications to help with disease diagnosis, testing drug efficacy, measuring disease progression, disease prognosis and drug safety among others.

**Figure 6:**
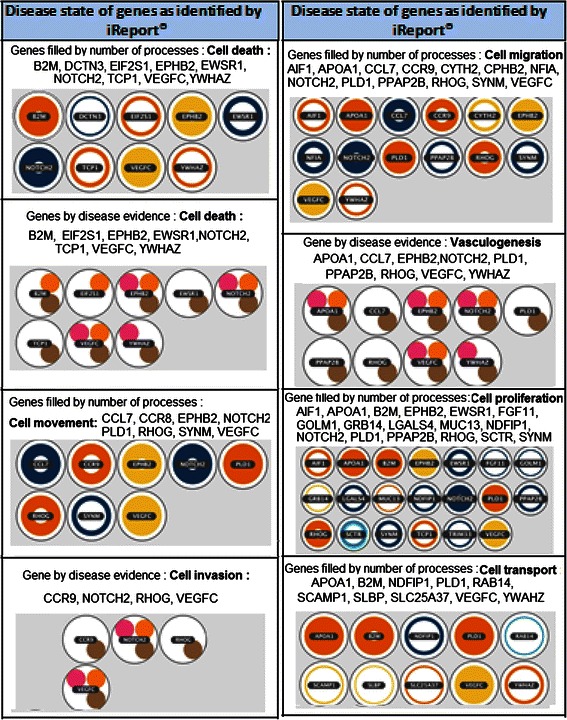
Hallmarks of cancer genes are listed here. The biological process and the genes that are related to some of the cancer hallmark processes such as cell cycle, death, movement, vasculogenesis, migration, proliferation, transport and invasion as identified by Ingenuity iReport®, are shown here. Cell proliferation and migration involves the largest number of genes and processes. The colour of the circle denotes expression level of each gene, with blue being the lowest and orange or red the highest. The disease state/evidence of genes are given by the smaller circles, where the small pink circles indicate that the gene is considered as a biomarker, an orange circle indicates that the gene is mutated in disease state, the brown circles indicate the level of expression, while the green circles (none here) indicate that the gene is a drug target. The gene names inside the coloured circles under disease state are listed in the second column.

Figure 6 gives the list of genes related to some of the cancer hallmark processes such as cell cycle, death, movement, vasculogenesis, migration, proliferation, transport and invasion as identified by Ingenuity iReport®. The nature of the disease evidence found for each gene is represented by different colours to indicate if they are biomarkers, mutations or differentially expressed genes where NOTCH2, EPHB2, YWHAZ, EPHB2, CCL7, B2M, APOPA1, SCAMP1, VEGFC, PPAP2B, mTOR, IGF and FGF are listed among others. Additional file 1: Table S9 summarizes the process counts, disease evidence and neighbour interactions for all the 52 genes that are of importance in the candidate gene set.

For the longest time traditional treatments for cancer centered on cytotoxic drugs and adjuvant therapies lacking precision to treating particular cancers; however, there is a tremendous shift towards creating therapies focusing on molecular targets that are rationally designed, aimed to bring greater efficacy with less harmful side effects.

## Conclusion

The proposed BCGA-ELM selects a minimum of 92 target genes (GCM) with a testing accuracy of 95.4%, which is between 21.6% and 38% higher than other results in literature for multi-class cancer classification. The molecular targets as identified in this study by the BCGA-ELM based multi-class algorithm has been shown to be reflective of the hallmarks of cancer [2]. We have used gene expression analysis to understand what molecular features might be specific to different types of cancers. The selected genes present hallmark features that contribute to processes that might initiate tumors, participate in cell migration and implement invasive properties that facilitate metastasis.

We hope that the BCGA-ELM algorithm can facilitate biomarker-driven integrated cancer research that can lead to a detailed understanding of the complexities of cancer. This understanding can lead to the development of drugs that are specific to each type of cancer that might be tailored to the needs of individual patients, leading to personalized medicine.

## Additional file

Additional file 1:
**Supplement-Multi-class-BCGA-ELM-based-classiffier-vasily-saras.**

## Abbreviations

IPA: Ingenuity Pathway Analysis
ELM: Extreme Learning Machine
GA: Genetic algorithm
BCGA-ELM: Binary Coded Genetic Algorithm with Extreme Learning Machine

## References

[1] Hanahan D, Weinberg RA (2000). The hallmarks of cancer. Cell.

[2] Hanahan D, Weinberg RA (2011). Hallmarks of cancer: The next generation. Cell.

[3] Ramaswamy S, Tamayo P, Rifkin R, Mukherjee S, Yeang C-H, Angelo M (2002). Multiclass cancer diagnosis using tumor gene expression signatures. Proc Natl Acad Sci U S A.

[4] Tapia E, Ornella L, Bulacio P, Angelone L (2011). Multiclass classification of microarray data samples with a reduced number of genes. BMC Bioinformatics.

[5] Abeel T, Helleputte T, Van de Peer Y, Dupont P, Saeys Y (2010). Robust biomarker identification for cancer diagnosis with ensemble feature selection methods. Bioinformatics.

[6] Dagliyan O, Uney-Yuksektepe F, IH K, Turkay M (2011). Optimization based tumor classification from microarray gene expression data. PLoS One.

[7] Holec M, Klema J, Zelezny F, Tolar J (2012). Comparative evaluation of set-level techniques in predictive classification of gene expression samples. BMC Bioinformatics.

[8] Tan AC, Naiman DQ, Xu L, Winslow RL, Geman D (2005). Simple decision rules for classifying humancancers from gene expression profiles. Bioinformatics.

[9] Chopra P, Lee J, Kang J, Lee S (2010). Improving cancer classification accuracy using gene pairs. PLoS One.

[10] Zhang J-G, Deng H-W (2007). Gene selection for classification of microarray data based on the bayes error. BMC Bioinformatics.

[11] Sun Y, Todorovic S, Goodison S (2010). Local-learning-based feature selection for high-dimensional data analysis. Pattern Analysis Machine Intell IEEE Transac.

[12] Cai H, Ruan P, Ng M, Akutsu T (2014). Feature weight estimation for gene selection: a local hyperlinear learning approach. BMC Bioinformatics.

[13] Wang L, Chu F, Xie W (2007). Accurate cancer classification using expressions of very few genes. IEEE/ACM Trans Computational Biol Bioinformatics.

[14] Hong JH, Cho SB (2008). A probabilistic multi-class strategy of one-vs.-rest support vector machines for cancer classification. Neurocomputing.

[15] Saraswathi S, Suresh S, Sundararajan N, Zimmermann M, Nilsen Hamilton M (2011). ICGA-PSO-ELM approach for accurate multiclass cancer classification resulting in reduced gene sets in which genes encoding secreted proteins are highly represented. Comput Biol Bioinform IEEE/ACM Transac.

[16] Chuang LY, Yang CH, Li JC, Yang CH (2012). A hybrid bpso-cga approach for gene selection and classification of microarray data. J Comput Biol.

[17] Piao Y, Piao M, Park K, Ryu KH (2012). An ensemble correlation-based gene selection algorithm for cancer classification with gene expression data. Bioinformatics.

[18] Wang H, Zhang H, Dai Z, Chen MS, Yuan Z (2013). TSG - a new algorithm for binary and multi-class cancer classification and informative genes selection. BMC Med Genet.

[19] Hall M, Frank E, Holmes G, Pfahringer B, Reutemann P, Witten IH (2009). The weka data mining software: An update. An update SIGKDD Explorations 11.

[20] Hoshida BJTPGTRMJPY (2007). Subclass mapping: Identifying common subtypes in independent disease data sets. PLoS One.

[21] Huang GB, Chen L, Siew CK (2006). Universal approximation using incremental constructive feedforward networks with random hidden nodes. IEEE Transac Neural Networks.

[22] Suresh S, Saraswathi S, Sundararajan N (2010). Performance enhancement of extreme learning machine for multi-category sparse cancer classification. EAAI 23.

[23] Holland HJ (1975). Adaptation in natural and artificial systems.

[24] Mitchell M (1989). An introduction to genetic algorithms, pp. 117{117. MIT press 25. Goldberg, D.E.: optimization and machine learning.

[25] Houck CR, Joines JA, Kay MG (1996). A genetic algorithm for function optimization: a MATLAB implementation. ACM Transac Mathematical Software 22.

[26] Pomeroy SL, Tamayo P, Gaasenbeek M, Sturla LM, Angelo M, McLaughlin ME (2002). Prediction of central nervous system embryonal tumor outcome based on gene expression. Nature.

