# Supplementary material for: Multi-class BCGA-ELM based classifier that identifies biomarkers associated with hallmarks of cancer
Source: BMC Bioinformatics. 2015 May 20;16:166. doi: 10.1186/s12859-015-0565-5 (PMC4448565; doi:10.1186/s12859-015-0565-5)

Supplementary materials : for the paper

Multi-class BCGA-ELM based classifier that identifies biomarkers associated with hallmarks of cancer

Vasily Sachnev [1,**,*], Saras Saraswathi [2,**], Andrzej Kloczkowski [2] and Suresh Sundaram [3]

[1]Department of Information, Communication and Electronics Engineering, Catholic University of Korea, Republic of Korea.
[2]Battelle Center for Mathematical Medicine at The Research Institute at Nationwide Children's Hospital; Department of Pediatrics, College of Medicine, The Ohio State University, Columbus, OH, USA.
[3] School of Computer Science, Nanyang Technological University, Singapore.
** Both authors contributed equally to this study.

Table S1 Samples used per class for training and testing from the GCM dataset.

	Cancer classes													
	Breast	Prostate	Lung	Colorectal	Lymphoma	Bladder	Melanoma	Uterus	Leukemia	Renal	pancreas	ovary	Mesothelioma	CNS
Training	8	8	8	8	16	8	8	8	24	8	8	8	8	16
Testing	3	2	3	3	6	3	2	2	6	3	3	3	3	4

Table S2: gives the full list of 92 features (selected from 16063 features in the GCM dataset) with their gene names, description, fold-change, cell location, type of molecule and biomarker properties.

ID	Symbol	Description	Location	Type(s)	Biomarker Application(s)
U19713_s_at	AIF1	allograft inflammatory factor 1	Nucleus	other	
Y09022_at	ALG3	ALG3, alpha-1, 3-mannosyltransferase	Cytoplasm	enzyme	
H69440_at	ANKRD13A	ankyrin repeat domain 13A	Plasma Membrane	other	
J00098_cds1_s_at	APOA1	apolipoprotein A-I	Extracellular Space	transporter	diagnosis, efficacy, safety, unspecified application
S57153_s_at	ARID4A	AT rich interactive domain 4A (RBP1-like)	Nucleus	transcription regulator	
R25326_at	ATP6AP2	ATPase, H+ transporting, lysosomal accessory protein 2	Cytoplasm	transporter	
S82297_at	B2M	beta-2-microglobulin	Plasma Membrane	transmembrane receptor	disease progression, efficacy, response to therapy, safety, unspecified application
D21878_at	BST1	bone marrow stromal cell antigen 1	Plasma Membrane	enzyme	
RC_AA293796_at	CCDC64	coiled-coil domain containing 64	Cytoplasm	other	
U45982_at	CCR9	chemokine (C-C motif) receptor 9	Plasma Membrane	G-protein coupled receptor	diagnosis, disease progression
W07209_at	CIB2	calcium and integrin binding family member 2	unknown	kinase	
RC_AA482126_at	CLDN3	claudin 3	Plasma Membrane	transmembrane receptor	diagnosis, unspecified application
T82181_at	CPN1	carboxypeptidase N, polypeptide 1	Extracellular Space	peptidase	
AA151569_s_at	CPT1C	carnitinepalmitoyltransferase 1C	Cytoplasm	enzyme	
M17316_at	CRYGA	crystallin, gamma A	Extracellular Space	other	
M19169_at	CST1	cystatin SN	unknown	other	
M85085_at	CSTF2	cleavage stimulation factor, 3' pre-RNA, subunit 2, 64kDa	Nucleus	other	
U59752_at	CYTH2	cytohesin 2	Cytoplasm	other	
W26651_s_at	DCTN3	dynactin 3 (p22)	Nucleus	other	
RC_AA252176_at	DDHD2	DDHD domain containing 2	Cytoplasm	other	

J02645_at	EIF2S1	eukaryotic translation initiation factor 2, subunit 1 alpha, 35kDa	Cytoplasm	translation regulator	efficacy
L41939_at	EPHB2	EPH receptor B2	Plasma Membrane	kinase	disease progression, unspecified application
U53786_at	EVPL	envoplakin	Plasma Membrane	other	
X66899_at	EWSR1	Ewing sarcoma breakpoint region 1	Nucleus	other	unspecified application
AA011452_at	EXOSC6	exosome component 6	Nucleus	other	
AA251957_at	FAM129A	family with sequence similarity 129, member A	Cytoplasm	other	
Z19675_at	FGF11	fibroblast growth factor 11	Extracellular Space	growth factor	
RC_AA454597_s_at	GOLM1	golgi membrane protein 1	Cytoplasm	other	diagnosis
RC_AA235707_at	GTF2IRD1	GTF2I repeat domain containing 1	Nucleus	transcription regulator	
RC_AA255617_at	GUCD1	guanylylcyclase domain containing 1	unknown	other	
M38193_rna1_s_at	GZMB	granzyme B (granzyme 2, cytotoxic T-lymphocyte-associated serine esterase 1)	Cytoplasm	peptidase	prognosis
RC_AA416963_at	H2AFJ	H2A histone family, member J	unknown	other	
M25079_s_at	HBB	hemoglobin, beta	Cytoplasm	transporter	
RC_D52154_s_at	IDS	iduronate 2-sulfatase	Cytoplasm	enzyme	
X16260_s_at	ITIH1	inter-alpha-trypsin inhibitor heavy chain 1	Extracellular Space	other	
AF004292_at	KBTBD2	kelch repeat and BTB (POZ) domain containing 2	unknown	other	
U11717_s_at	KCNMA1	potassium large conductance calcium-activated channel, subfamily M, alpha member 1	Plasma Membrane	ion channel	diagnosis
AB006781_s_at	LGALS4	lectin, galactoside-binding, soluble, 4	Extracellular Space	other	diagnosis
RC_AA463502_at	MFSD8	major facilitator superfamily domain containing 8	Nucleus	other	
W27054_at	MRTO4	mRNA turnover 4 homolog (S. cerevisiae)	Cytoplasm	other	
RC_AA053660_at	MUC13	mucin 13, cell surface associated	Extracellular Space	other	
Z48314_s_at	MUC5AC	mucin 5AC, oligomeric mucus/gel-forming	unknown	peptidase	efficacy
X17059_s_at	NAT1	N-acetyltransferase 1 (arylamine N-acetyltransferase)	Cytoplasm	enzyme	
RC_AA399445_at	NDFIP1	Nedd4 family interacting protein 1	Cytoplasm	other	

RC_AA437235_s_at	NFIA	nuclear factor I/A	Nucleus	transcription regulator	
RC_AA292931_at	NLRC5	NLR family, CARD domain containing 5	Cytoplasm	transcription regulator	
RC_AA521290_at	NOTCH2	notch 2	Plasma Membrane	transcription regulator	disease progression
R73982_at	PHLDB2	pleckstrin homology-like domain, family B, member 2	Cytoplasm	other	
U38545_at	PLD1	phospholipase D1, phosphatidylcholine-specific	Cytoplasm	enzyme	
AA461235_s_at	PNISR	PNN-interacting serine/arginine-rich protein	Nucleus	other	
U79294_at	PPAP2B	phosphatidic acid phosphatase type 2B	Plasma Membrane	phosphatase	
X74330_at	PRIM1	primase, DNA, polypeptide 1 (49kDa)	Nucleus	enzyme	
RC_AA446964_at	PSCA	prostate stem cell antigen	Plasma Membrane	other	unspecified application
RC_D19673_at	PTPN22	protein tyrosine phosphatase, non-receptor type 22 (lymphoid)	Cytoplasm	phosphatase	unspecified application
RC_AA403162_at	RAB14	RAB14, member RAS oncogene family	Cytoplasm	enzyme	
RC_AA411469_at	RASAL2	RAS protein activator like 2	Extracellular Space	other	
D31815_at	RGN	regucalcin (senescence marker protein-30)	Nucleus	enzyme	
X61587_at	RHOG	ras homolog family member G	Cytoplasm	enzyme	
X56932_at	RPL13A	ribosomal protein L13a	Cytoplasm	other	
X69150_at	RPS18	ribosomal protein S18	Cytoplasm	other	
M91391_at	RRP1B	ribosomal RNA processing 1 homolog B (S. cerevisiae)	Nucleus	other	
M84820_s_at	RXRB	retinoid X receptor, beta	Nucleus	ligand-dependent nuclear receptor	prognosis
RC_AA490323_at	SAE1	SUMO1 activating enzyme subunit 1	Cytoplasm	enzyme	
AF005037_at	SCAMP1	secretory carrier membrane protein 1	Cytoplasm	transporter	
U28281_at	SCTR	secretin receptor	Plasma Membrane	G-protein coupled receptor	
RC_AA167273_at	SDC3	syndecan 3	Plasma Membrane	other	
M68519_rna1_at	SFTPA2	surfactant protein A2	Extracellular Space	other	
RC_AA463886_s_at	SGTA	small glutamine-rich tetratricopeptide repeat (TPR)-containing, alpha	Cytoplasm	other	
U75679_at	SLBP	stem-loop binding protein	Nucleus	other	

C02500_s_at	SLC25A37	solute carrier family 25 (mitochondrial iron transporter), member 37	Cytoplasm	other	
U70867_at	SLCO2A1	solute carrier organic anion transporter family, member 2A1	Plasma Membrane	transporter	
RC_AA428995_at	STAG2	stromal antigen 2	Nucleus	other	
M97935_s_at	STAT1	signal transducer and activator of transcription 1, 91kDa	Nucleus	transcription regulator	diagnosis, efficacy, prognosis
AB002351_at	SYNM	synemin, intermediate filament protein	Cytoplasm	other	
X52882_at	TCP1	t-complex 1	Cytoplasm	other	
U90902_at	TIAM1	T-cell lymphoma invasion and metastasis 1	Cytoplasm	other	
RC_AA017254_at	TMEM196	transmembrane protein 196	unknown	other	
AA136360_at	TRIM33	tripartite motif containing 33	Nucleus	transcription regulator	
U04811_at	TRO	trophinin	Plasma Membrane	other	disease progression
AA259028_at	TUG1	taurineupregulated 1 (non-protein coding)	unknown	other	
U43142_at	VEGFC	vascular endothelial growth factor C	Extracellular Space	growth factor	diagnosis, disease progression, efficacy, prognosis
RC_AA007250_at	VWA8	von Willebrand factor A domain containing 8	Cytoplasm	other	
U79277_at	YWHAZ	tyrosine 3-monooxygenase/tryptophan 5-monooxygenase activation protein, zeta polypeptide	Cytoplasm	enzyme	diagnosis
U71601_at	ZKSCAN3	zinc finger with KRAB and SCAN domains 3	Nucleus	transcription regulator	
HG3342_HT3519_s_at	UnMapped				
AA071311_at	UnMapped				
AA452004_at	UnMapped				
J05032_at_2	UnMapped				
L76687_at_2	UnMapped				
X72308_at_2	UnMapped				
RC_AA024658_at	UnMapped				
RC_AA164851_at	UnMapped				

Table S3:lists the 52 differentially expressed genes (out of 92 genes selected from the GCM dataset), ordered according to fold-change, as identified byiReport ® to be involved in 25 pathways, 66 biological processes, 29 diseases and 3 interactions (Fig. S1).

	Symbol: Name	Fold Change	Molecular Function	Location	Path-ways	Processes	Diseases	Inter-actions
**1**	TUG1: taurineupregulated 1 (non-protein coding)	1.637	other	Unknown	0	0	0	0
**2**	RRP1B: ribosomal RNA processing 1 homolog B (S. cerevisiae)	1.564	other	Nucleus	0	0	0	1
**3**	PLD1: phospholipase D1, phosphatidylcholine-specific	1.409	enzyme	Cytoplasm	14	55	3	2
**4**	B2M: beta-2-microglobulin	1.387	transmembrane receptor	Plasma Membrane	11	48	16	1
**5**	PRIM1: primase, DNA, polypeptide 1 (49kDa)	1.34	enzyme	Nucleus	0	1	1	0
**6**	TCP1: t-complex 1	1.322	other	Cytoplasm	0	11	4	1
**7**	CCR9: chemokine (C-C motif) receptor 9	1.31	G-protein coupled receptor	Plasma Membrane	0	33	6	0
**8**	MUC13: mucin 13, cell surface associated	1.305	other	Extracellular Space	0	1	3	0
**9**	BST1: bone marrow stromal cell antigen 1	1.294	enzyme	Plasma Membrane	0	8	0	0
**10**	SLC25A37: solute carrier family 25 (mitochondrial iron transporter), member 37	1.29	other	Cytoplasm	0	3	0	0
**11**	AIF1: allograft inflammatory factor 1	1.282	other	Nucleus	0	11	1	0
**12**	APOA1: apolipoprotein A-I	1.276	transporter	Extracellular Space	8	66	29	2
**13**	RHOG: ras homolog family member G	1.247	enzyme	Cytoplasm	25	39	5	1

**14**	YWHAZ: tyrosine 3-monooxygenase/tryptophan 5-monooxygenase activation protein, zeta polypeptide	1.24	enzyme	Cytoplasm	9	8	9	3
**15**	MUC5AC: mucin 5AC, oligomeric mucus/gel-forming	1.239	peptidase	Unknown	1	0	1	0
**16**	ALG3: ALG3, alpha-1,3-mannosyltransferase	1.239	enzyme	Cytoplasm	1	0	1	0
**17**	EIF2S1: eukaryotic translation initiation factor 2, subunit 1 alpha, 35kDa	1.236	translation regulator	Cytoplasm	6	9	1	2
**18**	NAT1: N-acetyltransferase 1 (arylamine N-acetyltransferase)	1.223	enzyme	Cytoplasm	0	2	0	0
**19**	STAG2: stromal antigen 2	1.222	other	Nucleus	1	0	0	0
**20**	SLBP: stem-loop binding protein	1.221	other	Nucleus	0	1	0	0
**21**	NLRC5: NLR family, CARD domain containing 5	1.21	transcription regulator	Cytoplasm	1	8	0	1
**22**	EPHB2: EPH receptor B2	1.196	kinase	Plasma Membrane	4	47	9	0
**23**	CYTH2: cytohesin 2	1.177	other	Cytoplasm	0	11	0	0
**24**	SCAMP1: secretory carrier membrane protein 1	1.169	transporter	Cytoplasm	0	3	0	0
**25**	VEGFC: vascular endothelial growth factor C	1.159	growth factor	Extracellular Space	19	56	12	0
**26**	GRB14: growth factor receptor-bound protein 14	1.144	other	Plasma Membrane	1	2	0	0
**27**	EVPL: envoplakin	1.142	other	Plasma Membrane	0	1	0	0
**28**	RASAL2: RAS protein activator	-1.1	other	Extracellular Space	0	0	1	0

	like 2							
**29**	MFSD8: major facilitator superfamily domain containing 8	-1.143	other	Nucleus	0	0	1	0
**30**	CIB2: calcium and integrin binding family member 2	-1.16	kinase	Unknown	0	0	1	0
**31**	CPT1C: carnitinepalmitoyltransferase 1C	-1.169	enzyme	Cytoplasm	4	2	0	0
**32**	SCTR: secretin receptor	-1.178	G-protein coupled receptor	Plasma Membrane	0	12	1	0
**33**	RAB14: RAB14, member RAS oncogene family	-1.187	enzyme	Cytoplasm	0	1	0	0
**34**	GUCD1: guanylylcyclase domain containing 1	-1.201	other	Unknown	0	0	0	0
**35**	EWSR1: Ewing sarcoma breakpoint region 1	-1.245	other	Nucleus	0	16	7	0
**36**	CST1: cystatin SN	-1.251	other	Unknown	0	0	0	0
**37**	CCL7: chemokine (C-C motif) ligand 7	-1.253	cytokine	Extracellular Space	8	37	7	0
**38**	DARS: aspartyl-tRNAsynthetase	-1.286	enzyme	Cytoplasm	1	3	0	1
**39**	DDHD2: DDHD domain containing 2	-1.316	other	Cytoplasm	0	0	2	0
**40**	LGALS4: lectin, galactoside-binding, soluble, 4	-1.319	other	Extracellular Space	0	13	6	0
**41**	TRIM33: tripartite motif containing 33	-1.366	transcription regulator	Nucleus	0	5	0	0
**42**	CPN1: carboxypeptidase N, polypeptide 1	-1.442	peptidase	Extracellular Space	0	2	2	0
**43**	DCTN3: dynactin 3 (p22)	-1.453	other	Nucleus	0	3	0	0
**44**	NDFIP1: Nedd4 family interacting protein 1	-1.468	other	Cytoplasm	0	10	0	0
**45**	NOTCH2: notch 2	-1.516	transcription regulator	Plasma Membrane	2	45	20	0
**46**	GOLM1: golgi	-1.613	other	Cytoplasm	0	1	1	0

	membrane protein 1							
**47**	FGF11: fibroblast growth factor 11	-1.921	growth factor	Extracellular Space	5	1	0	0
**48**	PPAP2B: phosphatidic acid phosphatase type 2B	-2.093	phosphatase	Plasma Membrane	4	11	5	0
**49**	SGTA: small glutamine-rich tetratricopeptide repeat (TPR)-containing, alpha	-2.272	other	Cytoplasm	0	0	2	0
**50**	SYNM: synemin, intermediate filament protein	-2.616	other	Cytoplasm	0	14	0	0
**51**	NFIA: nuclear factor I/A	-2.699	transcription regulator	Nucleus	1	22	1	0
**52**	PHLDB2: pleckstrin homology-like domain, family B, member 2	-3.304	other	Cytoplasm	0	2	0	1

Table S4 Top Results based on Keywords show many of the pathways and diseases associated with the 92 genes selected by BCGA-ELM from the GCM dataset.

Top results based on Keywords	
ERK/MAPK Signaling (Pathway)	*Keyword:* **leukemia**
14-3-3-mediated Signaling (Pathway)	*Keyword:* **adenocarcinoma**
Ovarian Cancer Signaling (Pathway)	
Aryl Hydrocarbon Receptor Signaling (Pathway)	
Integrin Signaling (Pathway)	
Mitotic Roles of Polo-Like Kinase (Pathway)	
Cell Cycle: G2/M DNA Damage Checkpoint Regulation (Pathway)	
uveal melanoma (Disease)	*Keyword:* **melanoma**
Pancreatic Adenocarcinoma Signaling (Pathway)	*Keyword:* **Cancer**
Glioma Invasiveness Signaling (Pathway)	
NGF Signaling (Pathway)	
Colorectal Cancer Metastasis Signaling (Pathway)	
ILK Signaling (Pathway)	
GlioblastomaMultiforme Signaling (Pathway)	
Cdc42 Signaling (Pathway)	
mTOR Signaling (Pathway)	
Signaling by Rho Family GTPases (Pathway)	
PI3K/AKT Signaling (Pathway)	
Notch Signaling (Pathway)	
HMGB1 Signaling (Pathway)	
Bladder Cancer Signaling (Pathway)	
Role of Tissue Factor in Cancer (Pathway)	
Cardiac Hypertrophy Signaling (Pathway)	
Molecular Mechanisms of Cancer (Pathway)	
IL-17A Signaling in Airway Cells (Pathway)	*Keyword:* **lung**
Role of IL-17F in Allergic Inflammatory Airway Diseases (Pathway)	
maturity-onset diabetes of the young type I (Disease)	*Keyword:* **pancreas**
Pancreatitis (Disease)	

Table S5 The top 103 Pathways (20 DEGs) and 79 Diseases (29 DEGs) are listed below as discovered by iReport®

103 Pathways (20 DEGs)	79 Diseases (29 DEGs)
Clathrin-mediated Endocytosis Signaling	peripheral arterial occlusive disease
Antigen Presentation Pathway	cirrhosis of liver
ILK Signaling	invasion of tumor cells
IL-8 Signaling	primary biliary cirrhosis
mTOR Signaling	autosomal dominant amyloidosis
Bladder Cancer Signaling	congenital disorder of glycosylation type 1d
VEGF Signaling	combined apoA-I and apoC-III deficiency
Dolichyl-diphosphooligosaccharide Biosynthesis	anaphylotoxininactivator deficiency
Choline Biosynthesis III	autosomal recessive spastic paraplegia type 54
Pancreatic Adenocarcinoma Signaling	hypocholesterolemia
p70S6K Signaling	Alagille syndrome 2
LXR/RXR Activation	familial hypercatabolichypoproteinemia
Mitochondrial L-carnitine Shuttle Pathway	neuronal ceroidlipofuscinosis 7
Endoplasmic Reticulum Stress Pathway	Hajdu-Cheney syndrome
Gαq Signaling	Sjogren's syndrome
Lipid Antigen Presentation by CD1	growth of malignant tumor
Germ Cell-Sertoli Cell Junction Signaling	high density lipoprotein deficiency type 2
Ephrin Receptor Signaling	hypoplasia of lymph vessel
Triacylglycerol Biosynthesis	granulomatous hepatitis
Production of Nitric Oxide and Reactive Oxygen Species in Macrophages	uveal melanoma
IL-17A Signaling in Fibroblasts	ulcerated cutaneous xanthomatosis
Notch Signaling	invasion of glioblastoma cells
	Pancreatitis

tRNA Charging		
Role of PKR in Interferon Induction and Antiviral Response		neurological signs
Cell Cycle: G2/M DNA Damage Checkpoint Regulation		metastasis of sarcoma
Role of IL-17F in Allergic Inflammatory Airway Diseases		amyloidosis of liver
Signaling by Rho Family GTPases		maturity-onset diabetes of the young type I
Semaphorin Signaling in Neurons		amyloidosis of spleen
Phospholipase C Signaling		gastrointestinal tract cancer
Role of IL-17A in Arthritis		digestive organ tumor
Actin Nucleation by ARP-WASP Complex		cardiac fibrosis of left ventricle
Colorectal Cancer Metastasis Signaling		duplex kidney
Phospholipases		metastasis of lymph node
Myc Mediated Apoptosis Signaling		muscle cancer
Glioma Invasiveness Signaling		inflammation of myocardium
TREM1 Signaling		lymphatic metastasis
IL-17A Signaling in Airway Cells		growth of neuroectodermal tumor
ERK5 Signaling		blood protein disorder
Angiopoietin Signaling		transformation of kidney cells
Mitotic Roles of Polo-Like Kinase		surface area of atherosclerotic lesion
Chemokine Signaling		acute renal allograft rejection
Caveolar-mediated Endocytosis Signaling		tumorigenesis of ovarian cancer cell lines
Ephrin B Signaling		Huntington's Disease
VEGF Family Ligand-Receptor Interactions		metastatic colorectal cancer
Cytotoxic T Lymphocyte-mediated Apoptosis of Target Cells		synovial sarcoma
Regulation of Actin-based Motility by Rho		stenosis of pulmonary artery

Allograft Rejection Signaling	refractory schizophrenia
FGF Signaling	xanthomatosis
OX40 Signaling Pathway	colorectal cancer
FXR/RXR Activation	marginal zone cell lymphoma
Virus Entry via Endocytic Pathways	hemochromatosis
Communication between Innate and Adaptive Immune Cells	familial combined hyperlipidemia
Fcγ Receptor-mediated Phagocytosis in Macrophages and Monocytes	invasion of colon carcinoma cells
HMGB1 Signaling	germ cell cancer
Role of Pattern Recognition Receptors in Recognition of Bacteria and Viruses	growth of fibrosarcoma
Antioxidant Action of Vitamin C	hypospadia
IGF-1 Signaling	pigmented villonodularsynovitis
Amyotrophic Lateral Sclerosis Signaling	Acne
Cholecystokinin/Gastrin-mediated Signaling	invasion of cancer cells
Nitric Oxide Signaling in the Cardiovascular System	cholestasis
HIF1α Signaling	Usher syndrome
Rac Signaling	hypoplasia of heart
NGF Signaling	formation of gallstone
Sphingosine-1-phosphate Signaling	chronic colitis
Role of Tissue Factor in Cancer	jaundice
RhoA Signaling	familial hypercholesterolemia
14-3-3-mediated Signaling	nail disease
Atherosclerosis Signaling	lymphedema
PI3K/AKT Signaling	multiple myeloma
	peroxisomal acyl CoA oxidase deficiency

eNOS Signaling		
Ovarian Cancer Signaling		retinoblastoma
AMPK Signaling		mammary tumor
IL-12 Signaling and Production in Macrophages		immunodeficiency
Hepatic Fibrosis / Hepatic Stellate Cell Activation		follicular adenoma
Aryl Hydrocarbon Receptor Signaling		Marfan's syndrome
Epithelial Adherens Junction Signaling		plasma cell dyscrasia
Axonal Guidance Signaling		
GlioblastomaMultiforme Signaling		
Regulation of eIF4 and p70S6K Signaling		
CXCR4 Signaling		
Mitochondrial Dysfunction		
Tec Kinase Signaling		
Cdc42 Signaling		
Acute Phase Response Signaling		
Endothelin-1 Signaling		
Granulocyte Adhesion and Diapedesis		
RhoGDI Signaling		
PPARα/RXRα Activation		
Sertoli Cell-Sertoli Cell Junction Signaling		
Dendritic Cell Maturation		
Regulation of the Epithelial-Mesenchymal Transition Pathway		
EIF2 Signaling		
Agranulocyte Adhesion and Diapedesis		
ERK/MAPK Signaling		

Thrombin Signaling	
Integrin Signaling	
Actin Cytoskeleton Signaling	
LPS/IL-1 Mediated Inhibition of RXR Function	
Cardiac Hypertrophy Signaling	
Protein Ubiquitination Pathway	
Role of Macrophages, Fibroblasts and Endothelial Cells in Rheumatoid Arthritis	
Molecular Mechanisms of Cancer	
Protein Kinase A Signaling	

Table S6 The top 341 Processes involving 40 DEGs are listed, below as discovered by iReport® pathways in normal vs. cancer (while processing the 92 genes selected by BCGA-ELM from the GCM dataset)

341 Processes (40 DEGs)				
vasculogenesis		elongation of endothelial cells		apoptosis of myeloma cells
abnormal morphology of intraepithelial T lymphocytes		binding of actin		development of lymphatic system component
long term depression of hippocampus		adhesion of macrophage cancer cell lines		development of marginal-zone B lymphocytes
migration of endothelial cell lines		cell-cell adhesion of endothelial cell lines		synthesis of sphingomyelin
invasion of tumor cells		expansion of PBMCs		cell-cell adhesion of colon cancer cell lines
outgrowth of neurites		cell cycle progression of smooth muscle cell lines		quantity of effector memory T lymphocytes
Rac protein signal transduction		abnormal morphology of lateral cerebral ventricle		arrest in growth of lung cancer cell lines
migration of cells		maturation of germ cells		trafficking of antigen presenting cells
axonogenesis		invasion of brain cancer cell lines		size of epithelial cell lines
migration of brain cancer cell lines		mitosis of cervical cancer cell lines		abnormal morphology of bile duct
migration of tumor cell lines		migration of cervical cancer cell lines		growth of lymph vessel
cell movement of tumor cell lines		elongation of cells		quantity of CD4+ T-lymphocytes
phagocytosis of leukemia cell lines		function of CD4+ T-lymphocytes		mitogenesis of endothelial cells
expansion of vein		small GTPase mediated signal transduction		development of anterior commissure
synaptic transmission of mossy fibers		uptake of amino acids		morphogenesis of atrial septum
G1/S phase transition of bladder cancer cell lines		retraction of neurites		migration of kidney cell lines
induction of foam cells		development of alpha-beta thymocytes		efflux of cholesterol
efflux of alpha-tocopherol		transmigration of peripheral blood leukocytes		outgrowth of axons
migration of sling cells		recruitment of Th1 cells		homing of leukocytes
fusion of transport vesicles		uptake of cystine		colony formation of fibroblast cell lines
recruitment of sphingomyelin		inflammation of myocardium		development of vasculature
solubilization of phospholipid		growth of smooth muscle cell lines		chemotaxis of monocytes
cell flattening of cervical cancer cell lines		morphogenesis of optic nerve		cell spreading of brain cancer cell lines
outgrowth of spiral ganglion		orientation of axons		proliferation of eye cell lines
mitogenesis of retinal cells		abnormal morphology of lateral geniculate nucleus		exchange of GTP
desensitization of eosinophils		size of presynaptic terminals		formation of lymph vessel
folding of actin		cell division of fibroblast cell lines		release of cholesterol
quantity of CD1d restricted invariant T cell		depletion of cholesterol		shape change of basophils
recruitment of cholesterol		sprouting of mossy fibers		maturation of male germ cells
oxidation of 1-palmitoyl-2-linoleoyl-sn-glycero-3-phosphocholine		quantity of vitamin A		invasion of cancer cells
formation of sling cells		abnormal pruning of axons		development of thymocytes
onset of expansion of T lymphocytes		vacuolation of kidney cell lines		differentiation of epidermis
relocalization of alpha-beta thymocytes		metastasis of lymph node		morphogenesis of embryonic epithelial tissue
growth of endothelial tissue		elimination of lymphocytes		arrest in cell cycle progression of cervical cancer cell lines
removal of phosphatidylcholine		proliferation of cervical cancer cell lines		complexity of dendrites
secretion of lysophosphatidylcholine		formation of membrane ruffles		abnormal morphology of gamma-delta T lymphocytes
quantity of mononuclear leukocytes		branching of axons		cell viability of skin cell lines
adhesion of endothelial cell lines		dendritic growth/branching		development of B-1 lymphocytes
quantity of leukocytes		concentration of phospholipid		adhesion of skin cell lines
formation of dendritic spines		quantity of metal ion		chemotaxis of macrophage cancer

				cell lines
branching of blood vessel		neovascularization of eye		cell-cell adhesion of vascular endothelial cells
invasion of endothelial cells		transformation of kidney cells		exchange of GDP
cytotoxicity of cytotoxic T cells		depletion of phospholipid		synthesis of rRNA
differentiation of CD4+ T-lymphocytes		relaxation of pulmonary artery		size of kidney cell lines
development of B lymphocytes		chemotaxis of granule cells		invasion of cells
chemotaxis of cells		abnormal morphology of monocytes		ruffling
migration of adipoblasts		abnormal morphology of CD8-positive alpha-beta intraepithelial T-cell		exocytosis by cells
depletion of cholesterol ester		tumorigenesis of ovarian cancer cell lines		metabolism of phospholipid
metabolism of 4-aminosalicylic acid		import of heavy metal		cytolysis of lymphoma cell lines
granulomatous hepatitis		acute renal allograft rejection		bobbing of head
accumulation of actin stress fibers		movement of growth cone		quantity of intraepithelial T lymphocytes
apoptosis of lamina propria T lymphocytes		mitogenesis of melanoma cell lines		phagocytosis of leukocyte cell lines
extension of parallel fiber		induction of lamellipodia		migration of PBMCs
cleavage of L-lysine		transmission of phospholipid		transport of molecule
survival of thymic epithelial cells		conversion of protein		abnormal morphology of renal tubule
homing of Th17 cells		phagocytosis of bone marrow cell lines		formation of cellular protrusions
clearance of choline-phospholipid		lymphangiogenesis of lymph vessel		migration of cerebellar granule cell
invasion of glioblastoma cells		transmission of cholesterol		mobilization of neutrophils
epithelial-mesenchymal transition of adenocarcinoma cell lines		quantity of B-1 lymphocytes		migration of germ cells
lymphangiogenesis of ear		quantity of macrophages		mitogenesis of breast cancer cell lines
N-acetylation of 4-aminosalicylic acid		formation of neurites		migration of skin cancer cell lines
re-entry into cell cycle progression of cervical cancer cell lines		development of hematopoietic progenitor cells		cross-linkage of peptide
efflux of cholesterol ester		proliferation of neuronal cells		translocation of phospholipid
cell cycle progression of peripheral blood leukocytes		binding of leukocytes		abnormal morphology of dilated third cerebral ventricle
function of vestibular organ		abnormal quantity of lymphocytes		chronic colitis
development of callosal axon		abnormal morphology of Paneth cells		lack of CD8+ T lymphocyte
development of Th9 cells		epithelial-mesenchymal transition of pancreatic cancer cell lines		refolding of protein
shape of neutrophils		uptake of GABA		development of bile duct
cell movement of granule cells		migration of eye cell lines		stimulation of eosinophils
inflammation of heart		secretion of phosphatidylcholine		angiogenesis of skin
quantity of metal		efflux of phosphatidylcholine		patterning of somites
quantity of gamma-delta T lymphocytes		abnormal morphology of alpha-beta intraepithelial T cell		quantity of nitrite
accumulation of leukocytes		recruitment of monocyte-derived macrophages		synthesis of glutathione
chemotaxis of tumor cell lines		size of embryonic cell lines		quantity of mitotic spindle
trafficking of leukocytes		arrest in growth of neuroblastoma cell lines		activation of monocyte-derived dendritic cells
quantity of antigen presenting cells		sprouting		uptake of cholesterol ester
quantity of T lymphocytes		folding of protein		transport of vesicles
cleavage of arginine		organization of cytoskeleton		phagocytosis of cells
transport of cholesterol ester		morphology of endothelial tissue		migration of dendritic cells
chemokinesis of macrophage cancer cell lines		release of nitric oxide		arrest in interphase of tumor cell lines
cell-cell adhesion of lung cell lines		accumulation of T lymphocytes		binding of phagocytes
attachment of cervical cancer cell lines		quantity of CD8+ T lymphocyte		development of lymphoid organ
cytostasis of hepatoma cell lines		apoptosis of peripheral T lymphocyte		contractility of left ventricle
chemotaxis of lymphatic endothelial cells		abnormal morphology of nerve ending		cell death of mesothelioma cell lines
abnormal morphology of vestibular dark		import of cholesterol		activation of PBMCs

cells				
mingling of cells		invasion of colon carcinoma cells		binding of lipopolysaccharide
efflux of sphingomyelin		proliferation of cholangiocytes		development of corpus callosum
fusion of insulin secretory granules		lymphangiogenesis of cornea		proliferation of synovial fibroblasts
scattering of neural crest cells		removal of cholesterol		binding of mast cells
metaphase of bone cancer cell lines		long-term potentiation of mossy fibers		abnormal morphology of renal medulla
binding of cholesterol		granulopoiesis of cells		morphology of kidney
amyloidosis of spleen		arrest in G1 phase of brain cancer cell lines		inflammation of intestine
generation of marginal-zone B lymphocytes		abnormal morphology of hippocampal commissure		migration of fibroblast cell lines
development of lymphocytes		meiosis of spermatocytes		cell death of tumor cell lines
transport of heavy metal		translocation of cholesterol		arrest in growth of tumor cell lines
abnormal morphology of eye		hydrolysis of cholesterol ester		formation of dendrites
extension of neurites		interphase of tumor cell lines		transmigration of dendritic cells
quantity of lymphocytes		morphogenesis of epithelial tissue		gene silencing
quantity of phagocytes		function of lymphocytes		initiation of chemoattraction of cells
long-term potentiation of hippocampus		G1 phase of tumor cell lines		invasion of endothelial cell lines
abnormal morphology of renal cortex		homing of mononuclear leukocytes		quantity of Ca2+
proliferation of cells		uptake of D-glucose		binding of eosinophils
mobilization of cholesterol		morphology of mononuclear leukocytes		formation of microvilli
vacuolation of embryonic cell lines		attraction of cells		formation of membrane processes
vacuolation of epithelial cell lines		proliferation of endothelial cell lines		fusion of plasma membrane
metaphase of cervical cancer cell lines		hydrolysis of lysophosphatidic acid		secretion of histamine
transcription of RNA primer		abnormal morphology of optic tract		maturation of antigen presenting cells
elongation of cervical cancer cell lines		organization of intermediate filament cytoskeleton		function of T lymphocytes
size of brain cancer cell lines		initiation of synthesis of protein		development of synapse
quantity of apoptotic cardiomyocytes		morphogenesis of dendritic spines		abnormal quantity of phospholipid
transport of secretory vesicles		release of choline-phospholipid		lack of corpus callosum
secretion of sphingomyelin		abnormal morphology of CD8-positive alpha-beta cytotoxic T cells		catabolism of phospholipid
secretion of vitamin E		metabolism of carnitine		secretion of vesicles
				cell spreading
				transformation of fibroblast cell lines

Table S7 Top 25 Signaling and metabolic pathways in normal vs. cancer (while processing the 92 genes selected by BCGA-ELM from the GCM dataset)

	Pathways	pValue	Genes	DEGs
1.	Clathrin-mediated Endocytosis Signaling	0.002	APOA1, EPHB2, VEGFC, FGF11	4
2.	Antigen Presentation Pathway	0.005	B2M, NLRC5	2
3.	ILK Signaling	0.015	RHOG, PPAP2B, VEGFC	3
4.	IL-8 Signaling	0.015	RHOG, VEGFC, PLD1	3
5.	mTOR Signaling	0.016	RHOG, VEGFC, PLD1	3
6.	Bladder Cancer Signaling	0.026	VEGFC, FGF11	2
7.	VEGF Signaling	0.027	VEGFC, EIF2S1	2
8.	Dolichyl-diphosphooligosaccharide Biosynthesis	0.030	ALG3	1
9.	Choline Biosynthesis III	0.036	PLD1	1
10.	Pancreatic Adenocarcinoma Signaling	0.036	VEGFC, PLD1	2
11.	p70S6K Signaling	0.044	YWHAZ, PLD1	2
12.	LXR/RXR Activation	0.045	APOA1, CCL7	2
13.	Mitochondrial L-carnitine Shuttle Pathway	0.047	CPT1C	1
14.	Endoplasmic Reticulum Stress Pathway	0.049	EIF2S1	1
15.	Gαq Signaling	0.064	RHOG, PLD1	2
16.	Lipid Antigen Presentation by CD1	0.070	B2M	1
17.	Germ Cell-Sertoli Cell Junction Signaling	0.071	RHOG, PPAP2B	2
18.	Ephrin Receptor Signaling	0.086	EPHB2, VEGFC	2
19.	Triacylglycerol Biosynthesis	0.088	PPAP2B	1

**20.**	**Production of Nitric Oxide and Reactive Oxygen Species in Macrophages**	**0.090**	**APOA1, RHOG**	**2**
**21.**	**IL-17A Signaling in Fibroblasts**	**0.094**	**CCL7**	**1**
**22.**	**Notch Signaling**	**0.101**	**NOTCH2**	**1**
**23.**	**tRNA Charging**	**0.101**	**DARS**	**1**
**24.**	**Role of PKR in Interferon Induction and Antiviral Response**	**0.106**	**EIF2S1**	**1**

Table S8 Top molecules (biomarkers) implicated in Leukemia as discovered by IPA reports (while processing the 92 genes selected by BCGA-ELM from the GCM dataset), using cancer vs. normal data.

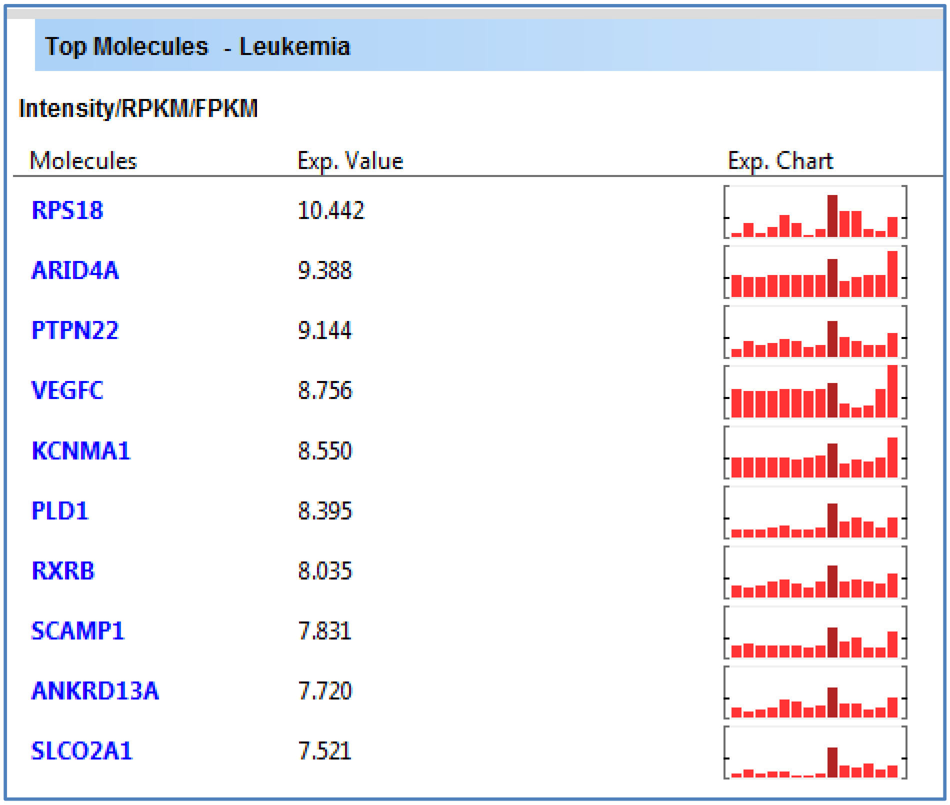

Top Molecules - Leukemia

Intensity/RPKM/FPKM

Molecules	Exp. Value	Exp. Chart
RPS18	10.442	
ARID4A	9.388	
PTPN22	9.144	
VEGFC	8.756	
KCNMA1	8.550	
PLD1	8.395	
RXRB	8.035	
SCAMP1	7.831	
ANKRD13A	7.720	
SLCO2A1	7.521	

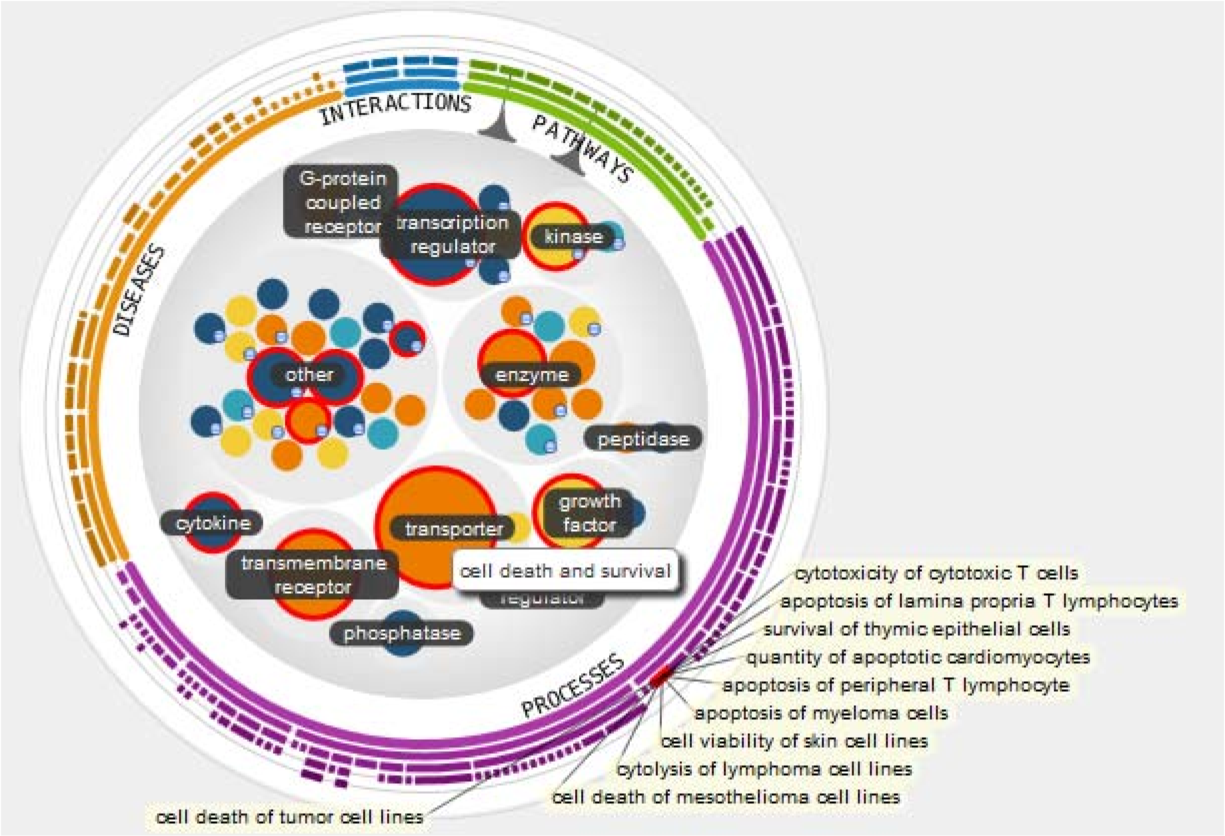

Fig. S1 Genes responsible for cell death and survival are given here as per iReport® , as discovered by IPA reports (while processing the 92 genes selected by BCGA-ELM from the GCM dataset), using cancer vs. normal data. Many adverse activities related to cell functions such as apoptosis are shown, where the different processes are listed and the genes involved are circled in red.}

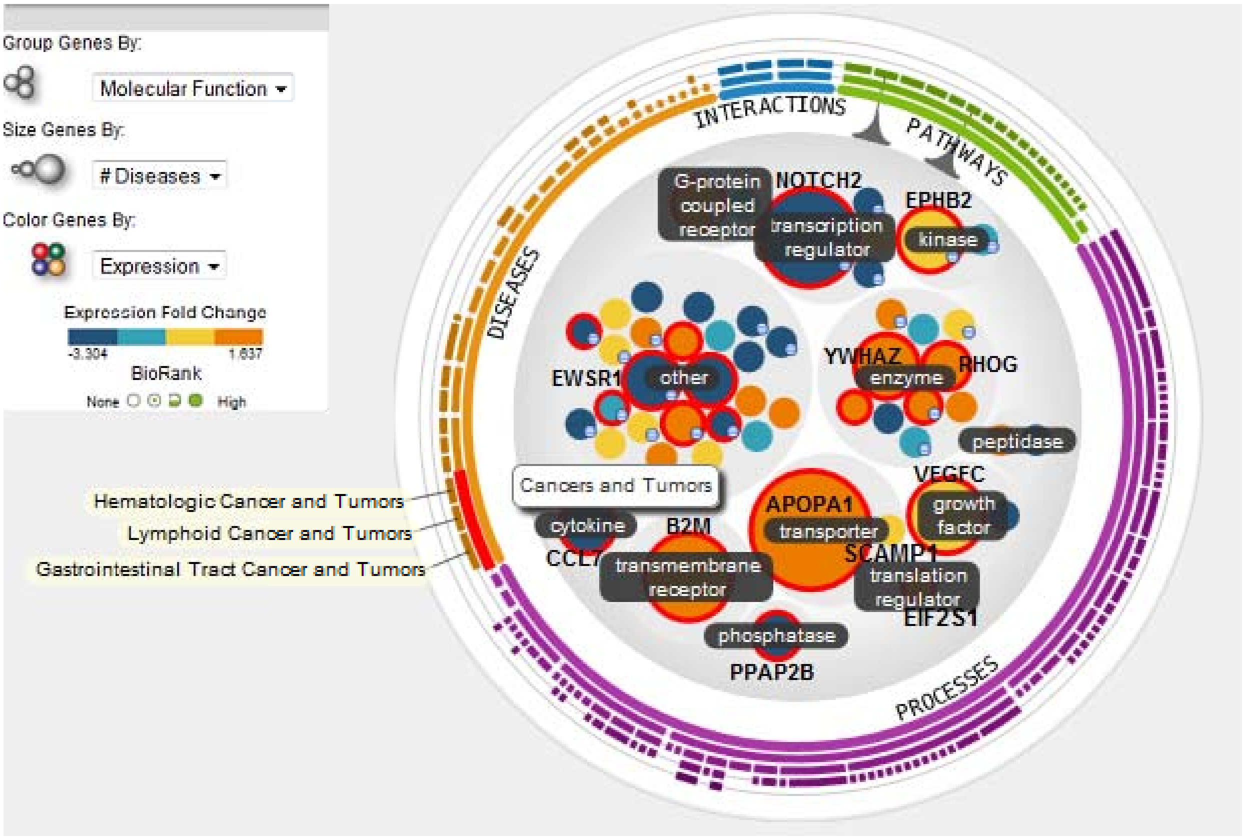

Fig. S2 Genes related to particular types of cancers that are highlighted are circled in red. APOPA1, NOTCH2, B2M and VEGFC seem to play major roles in these cancers. The full list of diseases with leading links are listed separately in Tables~S4 - S6. (as discovered by IPA reports , while processing the 92 genes selected by BCGA-ELM from the GCM dataset), using cancer vs. normal data.)

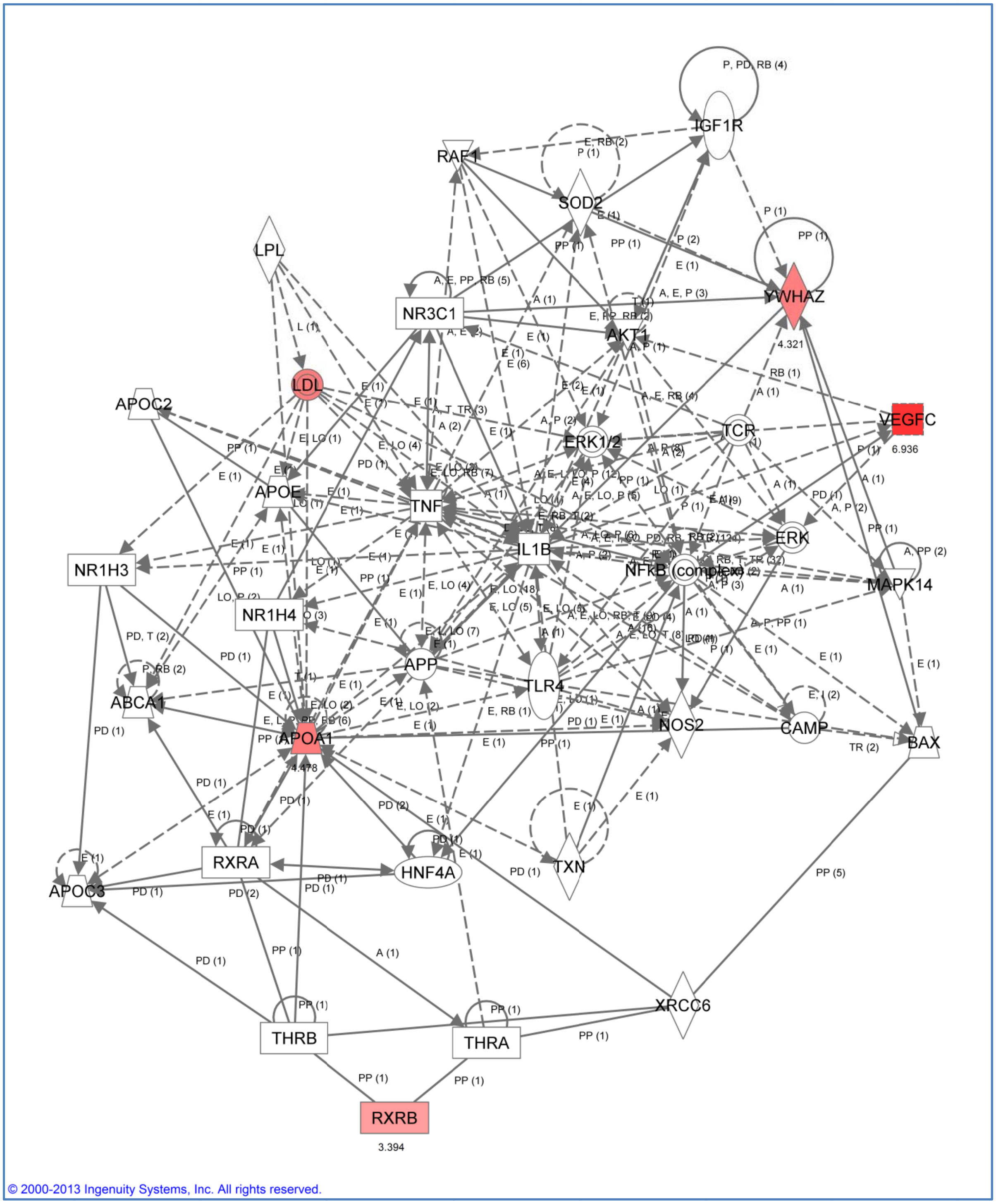

Fig. S3 A network implicated in breast cancer overlaid with biomarkers as discovered by IPA analysis, (while processing the 92 genes selected by BCGA-ELM from the GCM dataset), using cancer vs. normal data. It shows many of the top biomarkers such as APOPA1, YWHAZ, LDL, RXRB that are up regulated and highly involved in a complex network of processes.

Table S9 Hallmarks of cancer genes are given here as discovered by IPA analysis (while processing the 92 genes selected by BCGA-ELM from the GCM dataset), using cancer vs. normal data. The figure colors indicate isoforms, molecular function, number of diseases, processes, or interactions, cell location, mutation status drug target status, biomarker status, upstream interaction, downstream interaction, bi-directional interaction or binding interaction, connectivity rank score, biorank score, and total number of connected factors. In panel A, each gene is represented by a circle which is filled according to the number of processes it is involved in (40 DEGs). In panel B genes are denoted by disease evidence, where the small pink circles indicate that the gene is considered as a biomarker, an orange circle indicates that the gene is mutated in disease state, the brown circles indicate the level of expression, while the green circles indicate that the gene is a drug target (29 DEGs). Panel C indicates interactions between genes (20 DEGs). The filling of the genes show whether the interactions are downstream, upstream or binding. YWHAZ and EIF2S1 are interact in many processes and have a binding interaction while TCP1 has fewer processes.}

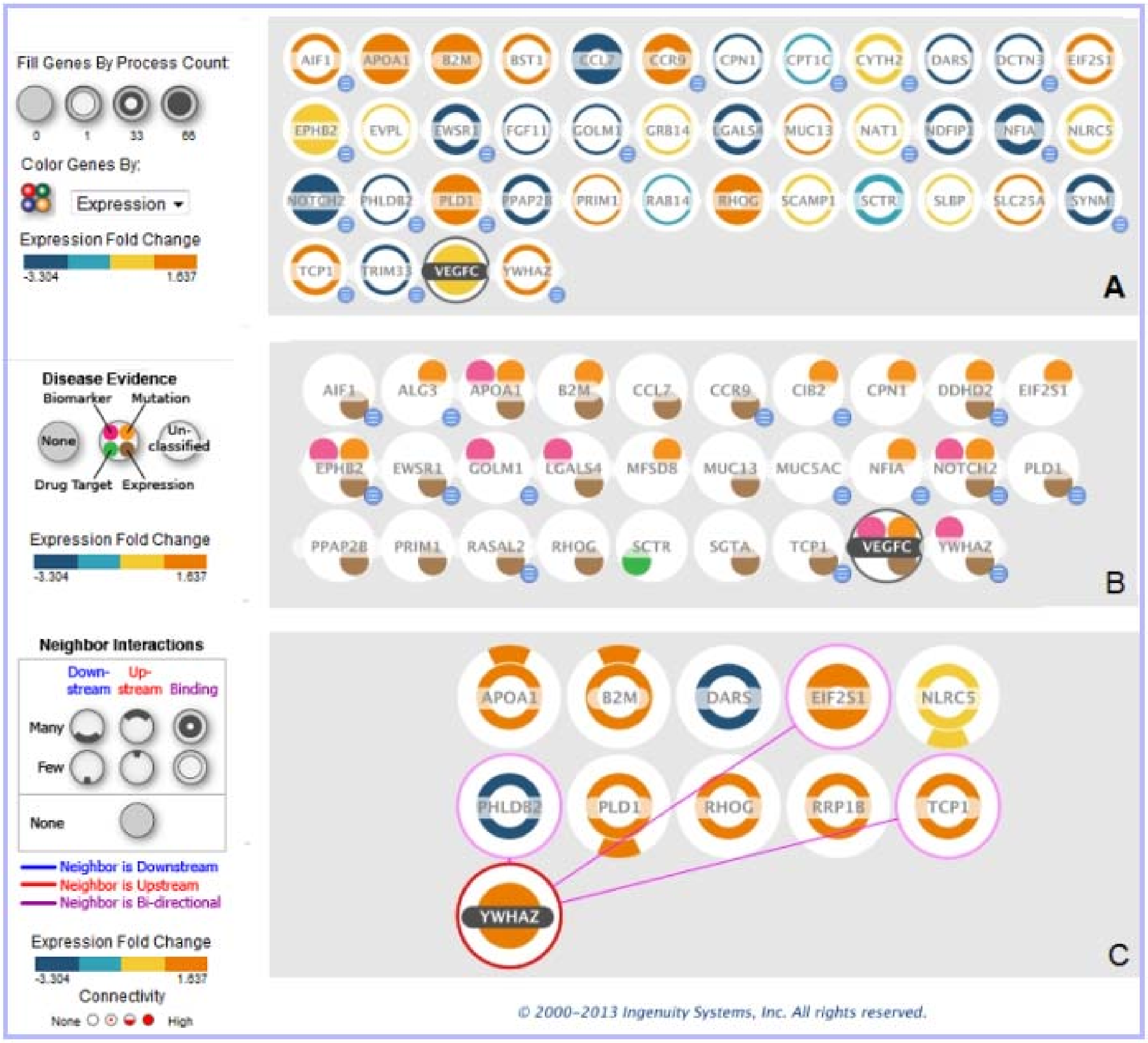

Supplement: Additional file 1: — Supplement-Multi-class-BCGA-ELM-based-classiffier-vasily-saras. [file 12859_2015_565_MOESM1_ESM.pdf]
